# Effect of deuterium irradiation on graphite boronized in the NSTX-U tokamak

**DOI:** 10.1038/s41598-019-38941-9

**Published:** 2019-02-21

**Authors:** F. Bedoya, J. P. Allain, F. J. Dominguez-Gutierrez, P. S. Krstic

**Affiliations:** 1 Plasma Science and Fusion Center, Massachusetts Institute of Technology, Cambridge, MA 02139 USA; 2 Department of Nuclear, Plasma, and Radiological Engineering, University of Illinois, Urbana, IL 61801 USA; 3 Max-Planck-Institute for Plasma Physics, Boltzmannstrasse 2, 85748 Garching, Germany; 4 Institute for Advanced Computational Science, Stony Brook University, Stony Brook, NY 11749 USA

**Keywords:** Surfaces, interfaces and thin films, Techniques and instrumentation, Computational science

## Abstract

Boronization has been used in the National Spherical Torus-Upgrade (NSTX-U) as first wall conditioning technique. The technique decreased the oxygen impurities in the plasma and the O% on the Plasma Facing Components (PFC) as measured with an *in-vacuo* probe. Samples were extracted from tiles removed from the tokamak for *post-mortem* and controlled studies. Ex-vessel low energy and fluence D_2_^+^ and Ar^+^ irradiations were characterized *in-situ* to elucidate surface evolution of a cored graphite sample with an intrinsic concentration of boron from a tokamak environment. In addition, quadrupole mass spectrometer measurements of emitted D-containing species during irradiation, indicate potential retention of D by the boronized graphite interface and correlated back to the surface chemistry evolution. Classical Molecular Dynamics (CMD) simulations were used to investigate the chemistry of the B-C-O-D system. The results suggest that boron coatings retain oxygen by forming oxidized boron states in the presence of deuterium plasmas and corroborate empirical findings. A four times increase in the O% of the boron coatings was observed following *in-situ* deuterium exposures, in contrast with a reduction of equal magnitude observed after Ar irradiations. These results illustrate the complex chemistry driven by energetic ions at the edge of tokamaks plasmas on the PFCs.

## Introduction

Tokamak devices remain as one of the most promising configurations to reliably harvest energy from fusion reactions through magnetic confinement of plasmas^1^. The density, temperature and energy confined dictate the plasma performance and greatly affect the overall performance of the machine^2^. Multiple studies have shown the strong effect that the Plasma Facing Components (PFC) and their chemical state have on plasma performance^3–6^. As a consequence, several methods of PFC conditioning have been developed and optimized to improve this performance^3,7,8^. The plasma assisted deposition of thin films of C, Si, Ti or B on PFC has been extensively used in different machines to improve the plasma behavior and protect the PFC^9^. In general, these coatings reduce physical and chemical sputtering and the amount of plasma impurities due to high sticking coefficients to the surface and oxide formation and adhesion^9–11^.

The National Spherical Torus Experiment Upgrade (NSTX-U) is a spherical tokamak with carbon-based PFC^12–14^. During the FY2016 experimental campaign, boronization was used as the main PFC conditioning technique in NSTX-U^13,15^. Boron was deposited using a DC glow of He and deuterated Trimethyl boron (d-TMB)^15^. This conditioning yielded boron rich coatings several nanometers thin that enabled plasma performance improvements shortly after their application^6,12^. Such coatings are applied with the intention of reducing the overall oxygen impurity content in the plasma by water and oxygen reduction at the boronized graphitic surface. However, the performance usually worsened with increasing plasma exposures requiring frequent subsequent boronizations. To understand the dynamic interactions of impurity fluxes to the boronized graphitic surfaces and their evolution under D plasma irradiation, *in-vacuo* characterization was conducted with the Materials Analysis Probe (MAPP) PMI (Plasma-Material Interactions) diagnostic and computational simulations of the B-C-O-D system dynamics and chemistry were carried out using Classical Molecular Dynamics (CMD) with reactive force field potentials (ReaxFF)^6,16,17^. This work complements these studies by examining NSTX-U tiles *post-mortem* and conducting controlled *in-situ ex-vessel* surface characterization studies, in close comparison with computed results (including oxygen and deuterium accumulation in the samples) to expand on MAPP results and provide a deeper fundamental understanding of D and impurity retention, surface chemistry and physics of boron-conditioned graphite.

*Post-mortem* analysis to investigate the accumulated effects of B conditioning and D plasma exposures during the campaign, and as part of the broader PFC investigation effort of the NSTX-U team, consisted of removing several tiles (exposed to multiple boronization and plasma discharges during the experimental campaign) from NSTX-U and core samples from them. This work reports the results of experiments performed under *ex-vessel-*controlled laboratory conditions in the IGNIS facility at University of Illinois, including low fluence deuterium and argon irradiation in combination with X ray photoelectron and gas spectroscopy analysis on one of the boronized graphite cored samples. Details on the experimental settings and measurements are provided in methods section. The CMD calculation uses the Reactive Force Field (ReaxFF) bond order potential adapted to the B-C-O-D system. Details of the calculations are depicted in the methods section as well.

In the same way, the results section is dedicated to the discussion of the experimental and modeling data. The same section presents our analysis of the results obtained in the laboratory using XPS and gas analysis in addition to the data obtained with the CMD simulations.

## Results

Previous studies on Li conditioning on ATJ graphite PFCs revealed the critical role the interaction between oxygen, lithium, carbon and deuterium has in the reduced fuel recycling and improved plasma performance^5,7,18^. Boron conditioning of PFCs also can lead to improved plasma performance. Reduced physical and chemical sputtering yields (compared with non-boronized carbon) or in the case of metal-based PFCs reduced high-Z erosion, added to boron’s oxygen gettering effect, are usually considered as the main reasons for its associated effect on the plasma^19–21^. CMD simulations combined with the *in-vacuo* MAPP data, shed new light on oxygen and deuterium retention mechanisms linked with boronization. To complement those results, a critical aspect of our scientific approach includes controlled *ex-vessel, in-situ* experiment to decouple the complexity of the tokamak plasma exposures. In this way, we can elucidate the dynamic effects where irradiation-driven mechanisms influence the surface chemistry and D retention in boronized graphite. These experiments were performed in the IGNIS facility, to improve the accuracy of these tests, we performed the irradiations and analysis on cores extracted from NSTX-U tiles, hence providing realistic samples of the complex surface chemistry found in the tokamak PFCs.

### D_2_^+^ and Ar^+^ irradiation of boronized graphite

**Figure 1:**
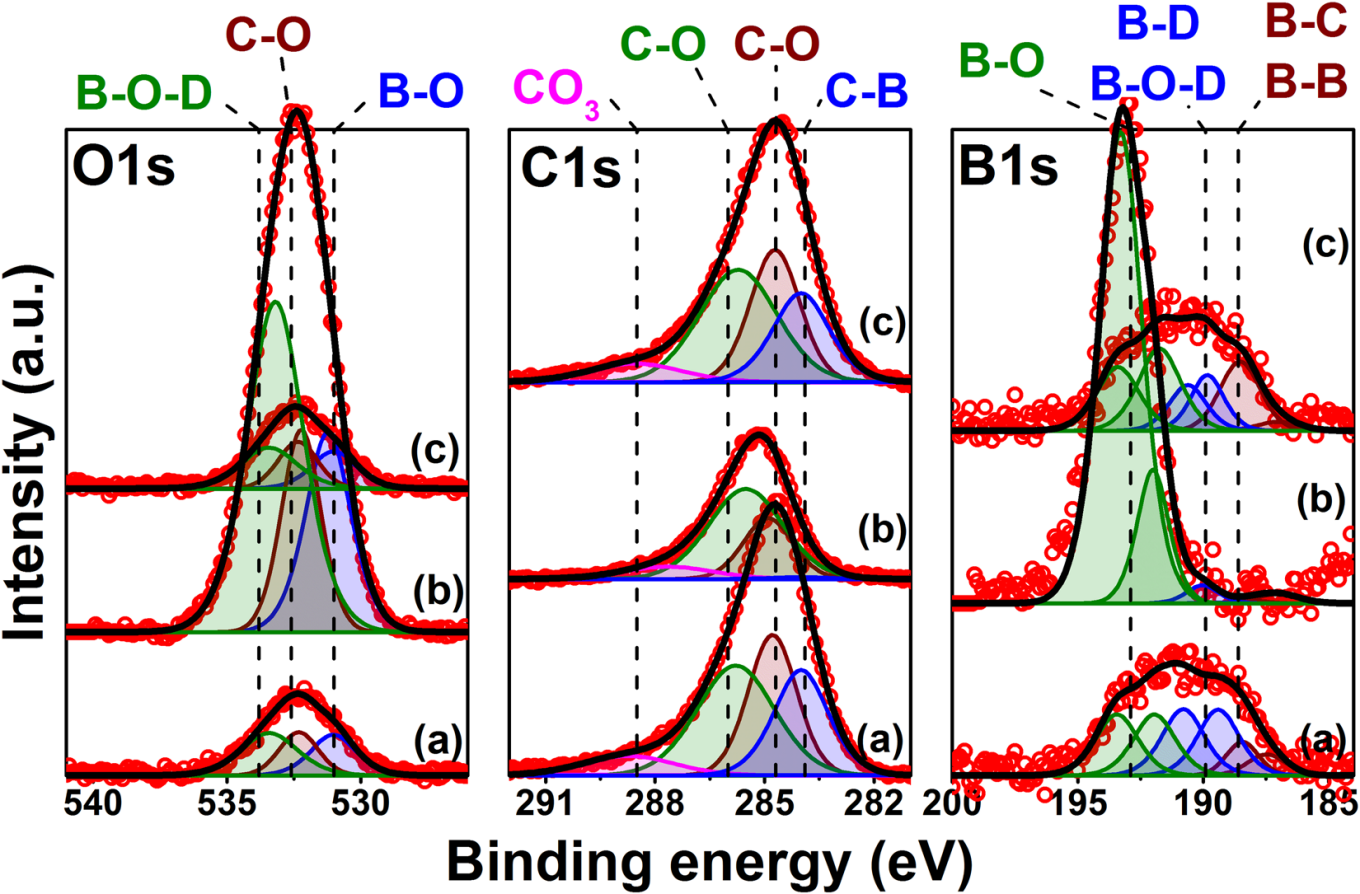
XPS spectra collected during the irradiation of the graphite sample. Each region is labeled on the top, (**a**) is the spectra collected following the Ar^+^ cleaning with 3.5 × 10^21^ m^−2^, (**b**) shows the XPS data after a D_2_^+^ irradiation with 6.0 × 10^20^ m^−2^ and finally (**c**) shows the data after the sample was exposed to additional Ar^+^ fluence of 2.0 × 10^21^ m^−2^.

Figure 1 shows the XPS data collected at three different stages of irradiation of the boronized graphite sample. Figure 1(a) shows the B1s, C1s and O1s regions after irradiation with Ar^+^ ions up to a fluence of 3.5 × 10^21^ m^−2^. Although the spectral XPS data are not included in this work, the surface chemistry of the sample previous to the irradiation with Ar^+^ was dominated by the presence of boron oxides clearly visible in the B1s and O1s region^22^. Similar chemical compositions were observed in samples exposed with the MAPP probe to boronizations and plasma exposures in NSTX-U^6,16^.

The Ar cleaning process relies on physical sputtering of the top monolayers of the material to initially remove contaminants attached to the surface during extraction, machining and transport of the cores, and, additionally to remove layers of the material revealing the chemistry as a function of depth. In this way, the trace shown by Fig. 1(a) shows the surface chemistry after the removal of roughly 100 nm of material, as determined with TRIM simulations of a boronized carbon surface irradiated with 1 keV Ar+. Although a BCA-based simulation code may not capture the complete physical conditions of the irradiated surface (e.g. accounting for surface chemistry effects and kinetic roughening), the results provide a broad estimate on the order of magnitude of the total erosion. The Ar cleaning process also mimics the inert gas irradiation process used in fusion devices such as NSTX-U with what is known as Glow Discharge Cleaning (GDC) typically conducted with helium low-temperature plasma.

The traces in Fig. 1(a) have a close resemblance to those obtained shortly after a boronization in NSTX-U as it was revealed by *in-vacuo* measurements done with MAPP^6^ i.e. low oxygen concentration and visible B-B and B-C interactions in the B1s region, including dominant B-D and B-O-D binding in the same region. Figure 1(b) shows the XPS data after two D_2_^+^ irradiations. The surface is now dominated by the presence of oxides as shown by the areas of the XPS envelopes in the B1s and O1s regions, which dramatically increased when compared with the same peaks in Fig. 1(a). Additionally, the position of the envelope in the B1s region in Fig. 1(a) shifted towards higher binding energy in Fig. 1(b) as a consequence of the increase in the B-O peak area and the decrease in the B-C and B-B peaks. This behavior was observed with MAPP in boronized surfaces exposed to plasmas in NSTX-U, as it also was in other controlled laboratory experiments^6,15,23,24^.

The oxygen gettering effect of boron coatings has been used to explain the improved plasma performance observed after boronizations in different tokamaks. Interestingly, the oxygen surface concentration increase on the sample was only observed following deuterium irradiation. Exposures to energetic argon ions did not induce any type of oxidation. Although, in the context of plasma performance in tokamaks, the impurity gettering effect of boron might be more relevant, the mechanisms associated with increased oxygen concentration and its potential effect on fuel recycling are not only related to thermodynamic reduction of oxygen from residual water vapor, but can also be driven energetically by the incident ion flux at the plasma-material interfaces. The thermodynamic oxidation kinetics on boronized graphite surfaces is mainly driven by diffusion-controlled rate laws, which for complex boronized graphite allotropes (i.e. such as high surface-area graphitic surfaces in NSTX-U), result in oxidation thresholds demonstrated by non-isothermal testing that vary between 0–10% degree of oxidation for temperatures between 400–700 °C^24^. For the operational conditions expected in NSTX-U with residual water pressure levels between 10^−5^ to 10^−4^ Pa, the expected Langmuir isotherm would result in less than 10% oxidation into B_2_O_3_.

Therefore, ion-induced segregation must be considered to justify the dramatic 400% increase of O on the surface measured with XPS *during* irradiation with D ions given the slow rate of oxidation kinetics by thermodynamic mechanisms alone. In the presence of energetic ions incident on the sample, species both mixed in the bulk and implanted via ambient water reduction of oxygen can diffuse towards the surface making the bulk an effective source of oxygen relative to the depth probed with XPS of nominally 2–5 nm. Ion-induced segregation is dominated by the relative values of enthalpy of formation of the molecules involved^25^. Consequently, in cases where the formation of impurity-incident ion compounds is more favorable than the bulk atom-incident ion compound, the diffusion of impurities towards the surface (defined by the implantation range of the ions) is possible^25–27^. For instance, in the case of graphite irradiated with D, the formation of D_2_O (−249.2 KJ/mol) and HDO (−245.37 KJ/mol) is more favorable than the formation of CD_4_ (−74.84 KJ/mol), as a consequence, the concentration of oxygen in carbon is reduced by irradiation with D^5^ and in fact, the D_2_O and HDO emission channels have been observed to be dominant during such irradiations^28^.

**Figure 2:**
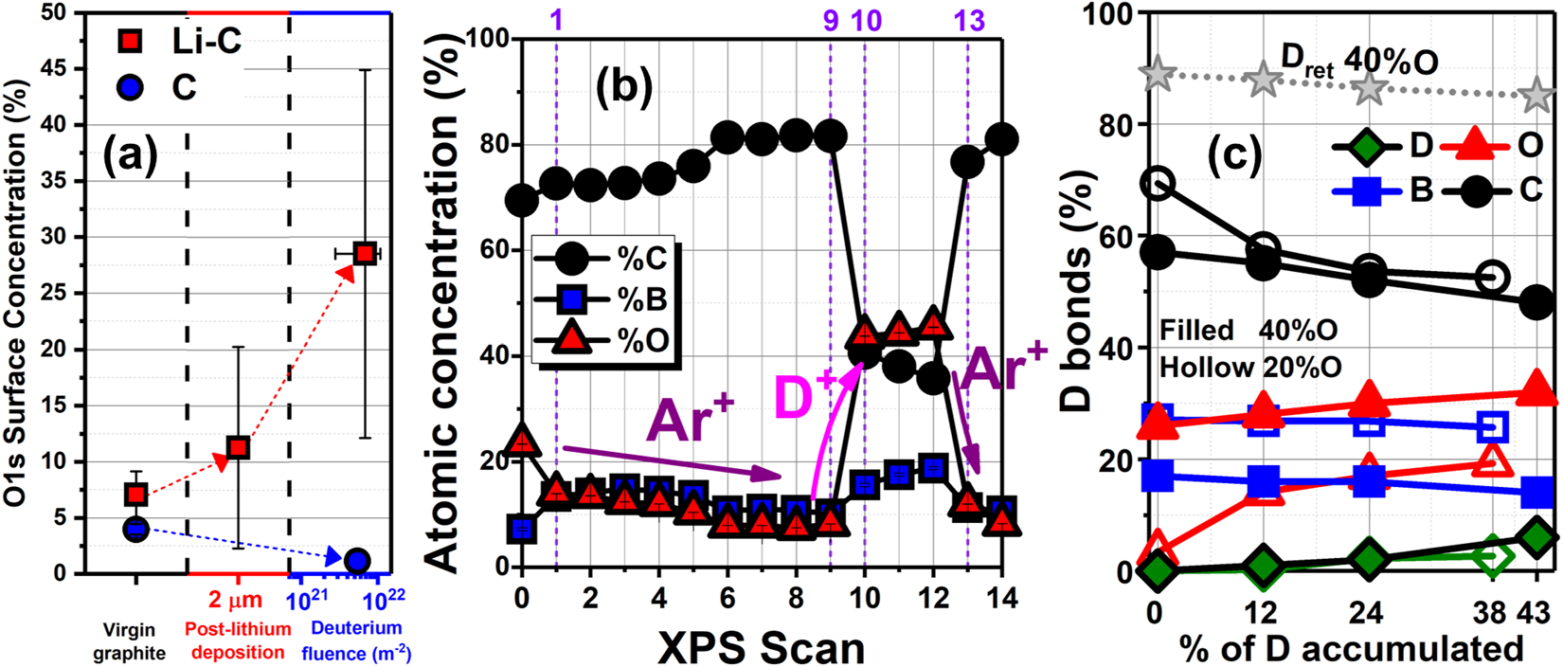
(**a**) Oxygen content of lithiaded and non-lithiaded graphite exposed to D_2_^+^ as shown in the x-axis (reproduced from^5^ with authorization from the publisher). (**b**) Atomic concentrations of B, C and O in the boronized graphite sample obtained with XPS. (**c**) Percentage of deuterium bonds by component as obtained with the CMD-Reaxff simulations for various accumulated deuterium concentrations, for two cases of ratio of concentrations of O:B:C, 40:20:40 (filled symbols) and 20:20:60 (hollow symbols)^18^. Total retention is showed for the 40:20:40 case.

In the context of PFCs in tokamaks a relevant example is that given by observations made in the Li-C-O-D system, as shown in Fig. 2(a)^5^. This plot displays the oxygen content, as measured with XPS, in lithiated and non-lithiated samples exposed to deuterium plasmas. In the case of Li irradiations with D plasmas, the formation of oxide (Li_2_O) is energetically more favorable as it is the emission of HDO and D_2_O^28^, hence the increase in the O% shown in Fig. 2(a). Similar mechanism could be involved in the evolution of the oxygen concentration in the B-C system reported in this work.

In general, the oxygen coverage evolves over time during exposure to boron conditioning and D plasma irradiations. The intrinsic coverage from boron oxide depositions are indicative of thermodynamic oxide reduction on the boron surface to approximately 25% (i.e. atomic percent) at the point “0” signaling the baseline surface oxygen coverage before argon cleaning as it can be seen in Fig. 2(b). This figure shows the concentrations of B, C and O plotted as a function of the XPS scan number, each point was obtained sequentially after an irradiation with deuterium or argon. The “0” trace is the pre-irradiation baseline as mentioned above, points 1 through 9 were obtained in between Ar^+^ irradiations, whereas points 10 to 12 were measured following D_2_^+^ exposures. Finally, points 13 and 14 were obtained after additional argon irradiations. For reference, the points 9, 12 and 14 correspond to the traces (a), (b) and (c) respectively in Fig. 1. The oxygen removal effect of Ar^+^ can be seen in the difference between points “0” and “1”, there, the O% changes from 25% to 15%. The effect of exposure to Ar^+^ irradiation can be further observed since as the data evolves from points “1” to “9”, the O% reduces from 15% to 5%, with a total fluence of 3.5 × 10^21^ m^−2^ Ar^+^ between the same points. This can be attributed to physical ion-induced desorption of oxygen during inert gas ion bombardment and physical sputtering of the oxide layers from the sample. This type of exposure mimics similar mechanisms to helium glow-discharge exposures in NSTX-U, in the sense that He and Ar are noble species that would not lead to particle-induced chemical reactions or would not act to catalyze reactions between the surface and the residual gases in the chamber.

The result of the next set of exposures are strikingly different. Three D_2_^+^ irradiations of equal fluence (2 × 10^20^ m^−2^) were performed following point “9” indicated between points “10” and “13”. Following the first deuterium plasma exposure an abrupt change in the concentration of oxygen is observed, going from 5% to 42%. This dramatic increase is attributed to two primary mechanisms: (1) the reduction of ambient water by energetic D_2_^+^ during irradiation and (2) the irradiation-driven migration of oxygen atoms to the surface. This result has also been observed when exposing lithiated graphite to deuterium irradiation as shown for comparison in Fig. 2(a). The post-lithium deposition on graphite yielded about 10% oxygen concentration, increasing to over 30% oxygen after D_2_^+^ irradiation. The figure also shows an interesting control result, where oxygen was removed by irradiation with deuterium ions, from a graphite surface with native oxide on its surface and no lithium deposition. This was expected due to the etching mechanisms, known to occur in hydrogen-carbon-oxygen interactions.

The increments of oxygen concentration from points “10” to “12” are considerably smaller close to approximately 3% after each irradiation, however still increasing with the reactive deuterium beam. When followed by an inert ion exposure with an Ar^+^ plasma, indicated from points “13” to “14”, the concentration of oxygen is dramatically reduced. Points 13 and 14 were obtained with data collected following 1 × 10^21^ m^−2^ Ar^+^ irradiations at each point. These results clearly show how in boronized graphite, oxygen adsorption can be only driven under certain conditions, in this case, in the presence of energetic D_2_^+^ ions.

### Atomistic simulations elucidate chemistry

CMD simulations performed on targets including B, C, O and D were performed using the approach described in the computational methods section. The results for the D chemistry in the B-C-O-D surface are summarized in Fig. 2(c), where the percentage of total D bonds is plotted as a function of total deuterium concentration for each one of the involved species, for two sets of atomic concentrations of the O, B and C species. The results were achieved by computing the nearest neighbors and their coordination numbers yielding the strongest favorable bond, as it is also explained in the computational methods section and similarly in ref.^17^. As discussed in ref.^18^, the data for the atomic concentration ratio for O:X:C = 20:20:60 show favorable retention by the XCOD matrices when compared with the rest of the configurations, i.e. when X = B the retention probability (per impact of 5 eV D) is 90%, while for the X = Li case, the same probability is 87%. These cases are followed by the XCO (86.5% for X = B and 84% for X = Li) and CO (~88%) cases. The lowest retention was obtained for the XC (~81% for X = B, and 79% for X = Li) and C (~77%) targets. For the case shown in Fig. 2(c) the D retentions decreases by more than 3% when D accumulation changes form 0% to the saturation value (43%) for the case with initial atomic concentrations of O:B:C = 40:20:40. According to the simulations, the mechanism of D retention in oxidized-deuterated boronized carbon, varies with increasing D accumulation. In the case of O:B:C = 20:20:60, as seen in Fig. 2(c), initially, B binds most of the incident D when the surface contains the same atomic concentrations of O and B (20%). Boron is more reactive than oxygen because of the so-called octet rule, i.e. a coordination number of four is preferred for B atoms, and in our simulations, even coordination numbers of five or six are sometimes possible, enabling larger number of D to bond to a B atom than to oxygen (with typical coordination number 2). Additionally, electron-withdrawing ligands on B such as O further increase D uptake on B. The role of B in the retention of D does not change with increasing D accumulation, although it is suppressed with increase of O atomic concentration. However, as the concentration of D increases, this D bonded to B slightly decreases and the additional deuterium attaches to the oxygen atoms in the target, reducing both the role of carbon and boron in the retention. In this way, the role of oxygen becomes more important when the concentration of D atoms is increased in the sample, and at 38% D concentration for O:B:C = 20:20:60 O binds almost the same amount as B, i.e. ~20%^17,29^. Interestingly, similar trends are also observed when O atomic concentration is set to 40% (and B 20%) i.e. O:B:C = 40:20:40. The difference is that the oxygen in this case plays a more significant role in bonding D even for 0% of accumulated D, while at 43% of D oxygen is as twice as effective in bonding D than boron. The increases and decreases of the roles of oxygen and boron, respectively, with accumulation of D show the same rate of change for both 40% and 20% of O (O:B:C = 20:20:60 and O:B:C = 40:20:40) cases.

### Correlating ion-induced desorption to surface chemistry *in-situ*

**Figure 3:**
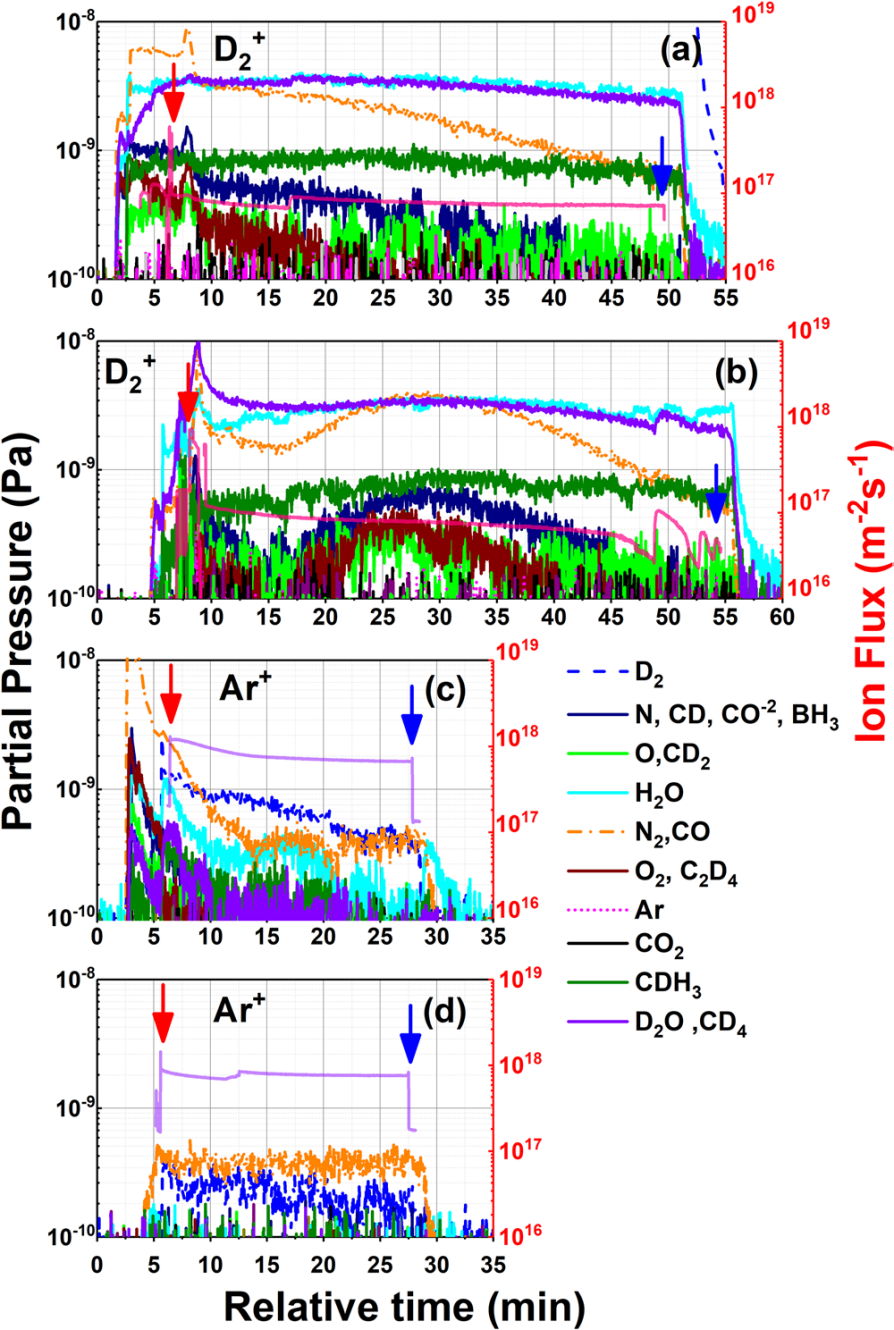
Residual gas analysis spectra collected during two deuterium and two argon sequential irradiations plotted as a function of time. The right axes refer to the partial pressure of each gas species as indicated by the labels. The left axes indicate the ion flux for D_2_^+^ in (**a**,**b**) (pink) and Ar+ in (**c**,**d**) (purple). The beginning of the irradiation is signaled with a red arrow, whereas the end is marked with a blue arrow. The irradiations shown in panels (a–d) occurred previous to the acquisition of the XPS data corresponding to the points 11, 12, 13 and 14 in the x-axis of Fig. 2(b) respectively.

The emission of different species from the sample, and their presence in the analysis chamber, in addition to other gas species, were monitored with a residual gas analyzer equipped with a quadrupole mass spectrometer. The ion flux, incident on the sample during irradiation was measured *in-situ*, with a pico-ammeter controlled via a serial port. Figure 3 shows the data collected with the RGA and the amp-meter during four different irradiations. Figure 3 shows the partial pressure of the different gases as a function of the irradiation time; the plot also displays the ion flux incident on the sample (pink line in 3(a) and 3(b) and purple line in 3(c) and 3(d)). Included in the figure, the label provides a list of the most probable fragments at each amu value.

The time coincidence of specific partial masses, measured by the mass spectrometer during irradiation, combined with *in-situ* surface chemistry characterization allows direct correlations of ion-induced desorption and ion-driven surface composition. Enabling analysis of the emitted species and correlating back to the surface chemistry and composition results in deciphering the dynamic changes that occur during energetic particle exposure. Although ultimately these mass spectrometry measurements can provide estimates of chemical sputtering, quantitative analysis is necessary and beyond the scope of this paper. Consequently, provided in Fig. 3 are the trends followed by the masses of the most probable fragments, considering the composition of the sample and the species used for irradiation.

Figure 3(a,b) show the data collected during two deuterium irradiations. The spectra are dominated by neutral species used to feed the plasma in this case deuterium (the partial pressure of D_2_ is around 10^−5^ Pa, not shown in the figures), additionally the partial pressure of water (18 amu) is relatively high compared with other masses.

In Fig. 3(b), nitrogen (N (14 amu) and N_2_ (28 amu)), oxygen (O (16 amu) and O_2_ (32 amu)) and the CD_4_ (20 amu) masses show a small increase in partial pressure starting around the 15 min mark. Temperature driven desorption (due to heating through irradiation) was discarded, since temperature measurements obtained under similar irradiation conditions yielded increments between 2–3 °C from room temperature.

Interestingly, the emission of these masses was only (or more strongly) observed during the irradiations with the reactive D_2_^+^ beam. The synchronized increase of masses 14 and 28 can be attributed to the emission of double charged CO, the formation of BH_3_, given boron’s three valence electrons, and to the presence of single charge CO respectively. These emission channels (CO in particular) have been previously observed in D irradiations of graphite and Li coated graphite. Considerable deuterium retention has also been observed in this system, in which the emission spectra during D_2_^+^ irradiations is usually dominated by HDO and D_2_O^28^, similar to that observed in Fig. 3(a,b), these emissions are attributed to the chemical sputtering from the D saturated surface^10,30^.

As mentioned before in the discussion of XPS data, these similarities suggest that dynamics similar to those observed in Li-C systems could be involved in the plasma-surface interactions in B-C systems i.e. irradiation-induced oxygen surface segregation and D retention and saturation, as also predicted by the simulations. This point is corroborated by combining both the *in-situ* XPS data that examines the surface chemistry evolution *and* the mass spectrometry of species released during irradiation. Although emission channels via chemical sputtering or chemically-enhanced desorption are certainly possible mechanisms, we speculate that the dominant emission of D atoms is controlled by the oxygen content on the surface as it reacts with D energetic particles near the surface. Ultimately the rate-limiting steps of surface recombination and emission will then dictate the retention of D in graphite.

Figure 3(c,d) show the partial pressure of gasses during two Ar^+^ irradiations. In these two cases, the spectra are also dominated by the neutral species inserted in the analysis chamber through the gas feed line i.e. Ar (argon partial pressure is ~10^−7^ Pa during the irradiations).

Remarkably, the figures show how the D_2_ mass is responsive to the ion flux measured on the sample, since, in both figures, this trace spikes up following the ion flux signal, again, in both cases around 5 minutes mark in the x-axis. In the same way, in these figures, the value of the partial pressure of D_2_ decreases steadily during both irradiations, starting close to 10^−9^ Pa in Fig. 3(c) and registering ~10^−10^ Pa at the end of the irradiation shown in Fig. 3(d).

The decreasing D_2_ and D_2_O partial pressure in Fig. 3(c,d) are evidence of deuterium retention by the oxide-boron layers on the graphite sample. As shown in Figs 1 and 2 and as discussed in the above D^+^ and Ar^+^ irradiation section, deuterium irradiation on boronized graphite, drives the oxidation of the surface, simultaneously, these freshly formed oxides retain upcoming deuterium molecules and atoms. There are three processes that are elucidated by the *in-situ* data during exposure of the PFC surfaces to energetic D_2_ particles. The XPS data shown above also show how irradiation with Ar^+^ removes the surface oxides along with retained deuterium as shown by the data in Fig. 3.

The point with these results is that realistic interfaces, found at the tokamak edge, will be an energetic-driven combination of oxygen and deuterium concentrations formed during plasma exposure and dictated by the surface chemistry presented in this work. Here, for the first-time we correlate *in-situ in-operando* mass spectrometry of the emitted products during irradiation with the time-dependent evolution of the surface chemistry.

## Discussion

We irradiated samples manufactured from tiles extracted from NSTX-U with D and Ar ions using low fluences at 250 eV/amu and 1000 eV respectively. To analyze the effect of these irradiations on the chemistry of the samples, we used *in-situ* XPS analysis in between the plasma exposures. Additionally, we performed mass spectrometry analysis species emitted during plasma exposures to correlated the irradiations and the surface characterization *in-operando* and *in-situ*. *In-situ* XPS data show that irradiation with argon removes contamination and oxides from the surface of the sample via physical sputtering, in this case, a total fluence of 3.5 × 10^21^ m^−2^ reduced the oxygen concentration in the sample around 15%. In contrast, the irradiation with deuterium ions, using a fluence approximately one order of magnitude less than that of Ar i.e. 6.0 × 10^20^ m^−2^, increased the oxygen concentration in the sample to 45%. We made similar observations in NSXT-U with the MAPP probe^6,22^, where progressive oxidation of boronized graphite was observed *in-vacuo*. Argon ions were later used on the sample again, removing the previously increased oxygen concentration. According to the mass spectrometry spectra collected, we deduce the oxidized boron coatings are able to retain deuterium under the conditions of our experiment. We conclude this since, the partial pressure of the *m/q* ratio corresponding to D_2_ and D_2_O show a strong dependence on Ar^+^ flux measured at the sample following two deuterium irradiations. Therefore, the mechanisms can be summarized as follows; initial irradiation with D_2_^+^ induces the oxidation of the boronized graphite (catalyzing residual water vapor adsorption and ion-induced segregation of retained oxygen towards the surface of the sample), similar to the case of Li coatings on carbon^5,31,32^. In the case of Li an increase of 18% in oxygen content was observed after D irradiation with and approximated fluence of 10^22^ m^−2 ^^5^, similarly, MAPP data obtained in NSTX-U showed remarkable oxidation of boron coatings on graphite (almost 25% increase in the oxygen concentration) following an average of 40 deuterium plasma discharges^6^. This oxidation promotes the bonding of D atoms on the surface via O-D interactions. Such mechanism, again in the both cases (B-C and Li-C), is responsible for the retention of D, especially at high deuterium fluences^17,33^.

The oxides can be later removed with Ar^+^, in such case the sputtered oxide layers leave the surface of the sample carrying the attached D atoms thus increasing their partial pressure in the vacuum. Since in this work the amount of implanted or deposited deuterium atoms is dictated by the relatively low fluence used, the partial pressure of this gas decreases almost linearly with time during the Ar irradiations, and is smaller in the second exposure than in the first one.

These measurements and analysis elucidate understanding of the mechanisms behind the improvements on plasma performance that B conditioning has in tokamaks, and, are congruent with the observations made *in-vacuo* with the MAPP probe. In this sense, the set of measurements presented here provides a confirmation under a more controlled, isolated experimental basis of the measurements obtained *on-site* with the MAPP.

CMD simulations show that deuterium retention chemistry in the BCOD system depends on the total amount of D implanted in the sample, as well as on the concentration of oxygen. According to the simulations, when the atomic concentration of oxygen is the same to that of boron (i.e. 20% or O:B:C = 20:20:60), oxygen only has a relevant role in trapping deuterium at high concentrations of D in the sample. However, if concentration of oxygen is set to 40% (O:B:C = 40:20:40), this is double the concentration of boron in the sample, oxygen suppresses the role of B in the D-bonding chemistry even for low concentrations of D, and this suppression increases with D accumulation.

Our results turn to be highly relevant in the assessment of conditioning methods and surface composition of PFC in tokamaks. In the case of NSTX-U, an increasing fluence of D ions produces increments in the concentration of O, as shown by our XPS data and in refs.^6,22^. This growth in the content of oxygen affects the sputtering properties of the PFC, since for instance, the sputtering yield varies from 0.122 for a target with 7.0% O to 0.161 for one with 40% O concentration when irradiated with 1.0 keV Ar^+^ ions as obtained using TRIM simulations, and, such proportionality can be also present in irradiations with different species. Furthermore, the yields for preferential sputtering predict 0.024 for B, 0.045 for C and 0.096 for O under low energy D irradiations irradiation^10^, as a consequence, the emission of oxygen (and any D bond to it) from the surface would decrease the plasma performance by increasing radiation loses (and increasing recycling). Such observations were also made in NSTX-U with the MAPP probe^6^.

## Methods

### Post-mortem analysis of NSTX-U tile surfaces

A *post-mortem* methodology was earlier designed by Taylor and Allain with NSTX staff for graphite-based reactive interfaces to provide surface characterization of the “realistic” material containing the overall plasma exposure history after thousands of shots in NSTX-U^32,34^. The methodology consists of coring samples from tiles extracted from the NSTX device at various locations known to have different PMI conditions (e.g. far scrape-off-layer, private flux region, etc.) transferred to high-resolution multi-analysis surface characterization tools. Although samples are exposed to atmosphere, Allain *et al*. demonstrated that a passivated layer effectively protects the *plasma-induced surface chemistry* below and can be excavated with energetic Ar^+^ ions carefully sequenced between surface composition measurements *in-situ*. Therefore, one can distinguish with high reliability the passivated surface region compared to the active region of interest in the relevant core-level XPS spectra. Here we define “*in-situ”* in terms of characterization of the sample surface *in place* of surface irradiations.

Three tiles were removed from different locations of NSTX-U (Inner Lower Divertor (ILD), Outer Lower Divertor (OLD) and Center Stack Shoulder (CSS)) at the end of the experimental campaign. PFC in NSTX-U are manufactured with ATJ (lower divertor tiles) and POCO (center stack shoulder) graphite. These locations were intentionally selected to investigate PFC at locations subjected to different plasma conditions during the experimental campaign. In this case, the OLD is the common location of the Outer Striking Point (OSP), and is, in general, considered as the main source of impurities in diverted tokamaks. In the same way, the CCS location is the place where the Inner Striking point ISP is usually placed, and, according to multiple studies in material migration, is the destination of most of the eroded particles. Lastly, the ILD usually exhibits a high concentration of gas neutrals and low ion fluxes^35,36^.

In the development of the campaign the tiles were exposed to multiple boronizations under different configurations (gas injectors and electrodes as shown in ref.^15^) and conditions, in addition to thousands of deuterium plasma shots, Helium Glow Discharge Cleaning (HeGDC) procedures and additional plasma glows with Ne. Details on *in-situ* chemical analysis of samples exposed to boronizations in NSTX-U and on the procedure itself can be found in refs^6,15^.

Cores with 1 cm in diameter were extracted from each tile for different characterizations e.g. SIMS, XPS, RBS. *Post-mortem* characterization of one core from each tile revealed a lower concentration of oxides in the CSS sample than those from the OLD or ILD after cleaning with Ar^+^ ions. As a consequence, this sample was also used for further D_2_^+^ irradiations and the measurements presented in this work were obtained with one of the CSS cores.

### *In-situ* surface chemistry characterization

**Figure 4:**
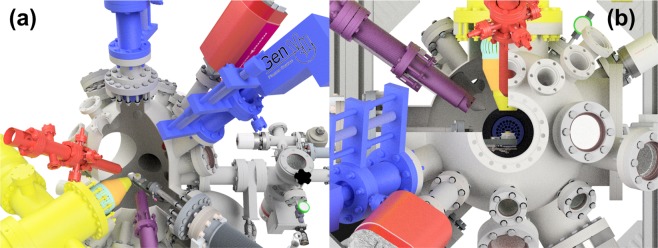
Renderings of the IGNIS facility at the University of Illinois, (**a**) shows the sample in the irradiation position, the blue elements are Tectra sources, the red elements are low flux ion guns for ion-based diagnostics, (**b**) shows the sample in the XPS analysis position, here the yellow element is the energy analyzer while the XRS is shown in purple. CAD created by the authors.

The Ion-Gas and Neutrals Interactions with Surfaces (IGNIS) facility is an *in-situ, in-operando* surface modification and characterization facility at University of Illinois. A schematic representation of IGNIS is shown in Fig. 4. The system exposes samples between low and moderate levels of hydrogen isotope and helium fluences and includes several surface characterization techniques including: X-ray Photoelectron Spectroscopy, Ion Scattering Spectroscopy and Thermal Desorption Spectroscopy.

One unique advantage of IGNIS, compared with similar facilities is the possibility to characterize the chemistry of a surface during irradiation under harsh environmental conditions of temperature (e.g. 77–1000 K), energetic particle bombardment (10–1000 eV) and high pressure (due to an additional background gas or to a high working pressure to the presence of gas neutrals) close to 0.1 Pa, mimicking plasma-material interaction in fusion devices. This is possible thanks to an advanced differentially pumped hemispherical analyzer (modified SPECS 150 NAP). The design of the analyzer includes two differential pumping stages, each one with a 600 L/sec turbo-molecular pump. These stages are separated by a set of mechanical diaphragms that isolate the analysis chamber from the section of the analyzer that contains the Micro Channel Plate detector.

This configuration can lead to pressure differences close to four orders of magnitude between the analysis and the MCP chambers. Additionally, the analyzer has an ultimate energy resolution of 2.5 meV^37^. To perform high-pressure XPS, IGNIS consists of a water-cooled dual anode SPECS XR-50 high-pressure X-Ray Source (XRS), similarly to the analyzer, differentially pumped to perform at pressures as high as 0.1 Pa. To meet high-pressure requirements in the XRS the volume that contains the thoriated W filaments is isolated from the IGNIS analysis chamber^37^. For the irradiations included in this work, we used a Tectra Gen II plasma source. This is a microwave source capable of operating with noble and reactive gases^38^. Ion fluxes close around 10^18^ m^−2^ s^−1^ with Ar^+^ and 10^15^ m^−2^ s^−1^ for D_2_^+^. The vacuum quality and the presence of residual gas impurities are monitored in IGNIS with a high-pressure Residual Gas Analyzer (RGA) (Inficon Transpector® XPR3) applying quadrupole mass spectrometry. This device can operate at pressures as high as 0.1 Pa and record the m/q signals from 2 to 100 amu with a resolution of 1 amu^22^.

**Figure 5:**
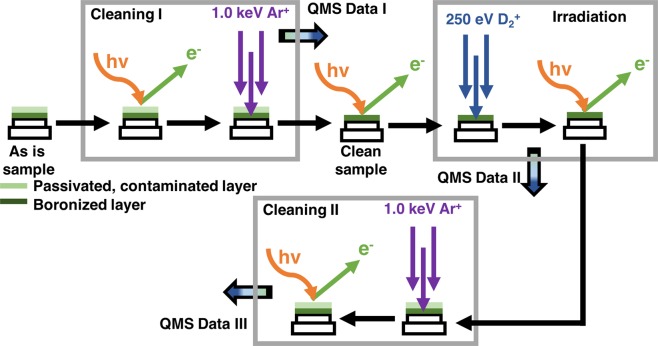
Schematic representation of the experimental procedure. XPS and QMS data was acquired during the different stages i.e. two Ar^+^ cleaning and one D_2_^+^ irradiation steps.

The sample was loaded in a custom designed holder and pumped down to 10^−5^ Pa for its insertion into the analysis chamber. The IGNIS analysis chamber usually has a base pressure of 10^−6^ Pa with a partial pressure of water close to 10^−9^ Pa. Initial XPS baseline spectra were then collected, this group of data includes a survey scan and three region scans (B1s, C1s and O1s). Following the first characterization several irradiations were performed with the Tectra source using 1 keV Ar^+^. The first irradiations totaled 3.5 × 10^21^ m^−2^ fluence and were done in incremental steps, starting from 10^17^ m^−2^ and with a final value after the last irradiation of 10^21^ m^−2^. Additional XPS data groups were collected in between irradiations including the same regions mentioned above. Following the Ar^+^ treatment three D_2_^+^ irradiations of 2.0 × 10^20^ m^−2^ (250 eV/amu) were performed on the sample. As before, XPS data were collected at the end of each irradiation. Finally, the sample was exposed to two more Ar^+^ irradiations of 10^17^ m^−2^ fluence each, XPS data was again measured following each exposure. A quadrupole mass spectrometer (QMS) was used during all the irradiations to collect data that would reveal possible emission of species from the sample when exposed to Ar^+^ or D_2_^+^. The QMS was set to follow amongst others D_2_, H_2_O, O, O_2_ and D_2_O (4, 18, 16, 32 and 20 amu). Unfortunately, masses 3 and 19 amu (HD and HDO) were not recorded during the experiment, thus making it challenging to quantify the total desorbed D (emission through these two channels usually amounts to over 50% of the total absorbed D in ATJ graphite^28^). To evaluate the dependence of each one of the m/q values on the ion flux, the current induced by the ion beam was measured with a pico-amp meter connected in series to the sample manipulator. Each partial pressure in time could be then compared with the same variation of the ion flux. The experimental procedure is shown schematically in Fig. 5.

### Computational methods

Our atomistic simulations are based on Classical Molecular Dynamics, with use of the Reactive Force Field (ReaxFF) Bond Order potential^39,40^ adapted to the B-C-O-D mixture^40–42^ of materials and implemented in the Large Scale Atomic/Molecular Massively Parallel Simulator (LAMMPS)^43^. The ReaxFF method applies the Electronegative Equalization Method (EEM)^44,45^ at each time step. This is particularly important in the presence of mutually polarizable materials such as boron, and oxygen. We verified the CMD calculations by Quantum-Classical Molecular Dynamics (QCMD), using approximate Self Consistent Charge Tight Binding Density Functional Theory (SCC-DFTB)^46^ to compute electronic motions in the adiabatic limit of nuclear motion. The procedure for formation, annealing and optimization of the computational cell is explained in detail in refs^10,11,33^.

We use a computational cell of about 400 atoms for various amorphous mixtures of B, C, and O. Boronized and oxidized systems are created by random substitution of carbon atoms to the desired atomic concentrations, estimated by the experiment to be atomic composition of (20, 40, 40) % for B, C, O respectively. The mixtures are then repeatedly annealed and finally thermalized to 300 K and energy optimized with periodic boundary conditions in the x-y directions, following the procedure of Krstic *et al*.^33,47^.

**Figure 6:**
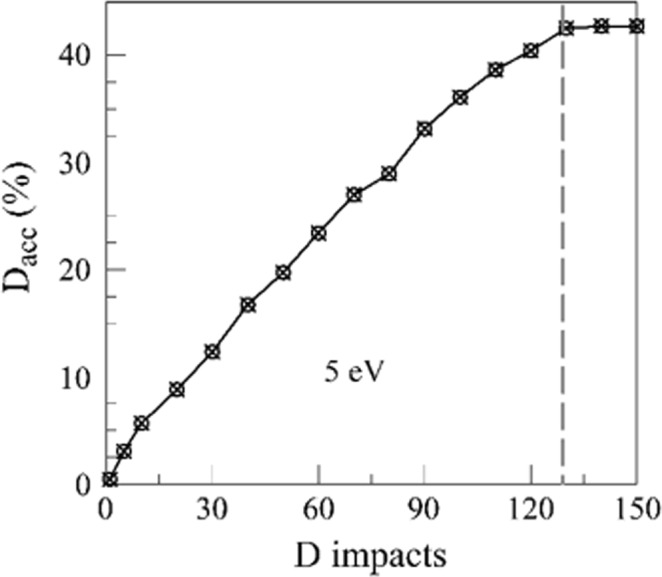
Percentage of implanted D as function of number of D impacts needed to prepare the BCO:D surface at 5 eV. The saturation of D is reached at 43% of D.

This procedure allows the formation of complexes with the chemical properties studied in this paper. Additional reformation of the chemical complexes evolves upon bombardment with D atoms, as explained below. To consider typical experimental conditions of the accumulated deuterium, the B-C-O cells of total of 400 atoms were prepared by cumulative bombardment with 5 eV D atoms, reaching various atomic concentrations of D. The penetration of D at 5 eV was distributed in depth up to 8 Å, and therefore the D atomic concentrations $\frac{{n}_{D}}{{n}_{C}+{n}_{B}+{n}_{O}}\times 100 \%$ were defined for the part of the computation cell from the top surface to 8 Å depth. The highest accumulated concentration of D reached was 43%, and this corresponds to the D saturation at the considered impact energy (Fig. 6).

**Figure 7:**
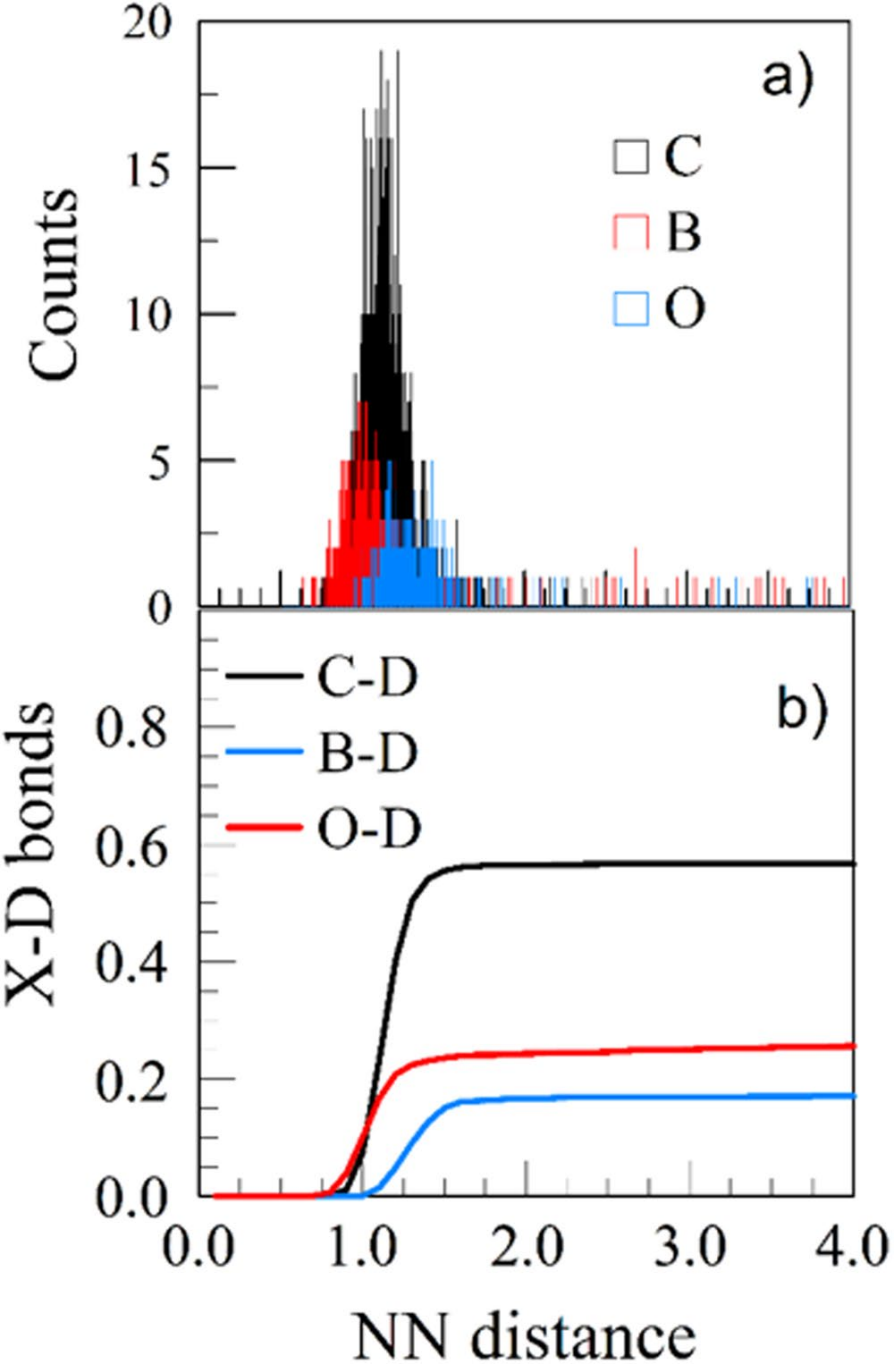
(**a**) Histogram and (**b**) cumulative distribution of the X-D (X = B,C,O) NN distances of the pristine BCO surface replicas bombarded over 3000 times with 5 eV D. Cumulative distribution functions show the percentages of X-D bonds, 57% of C-D, 26% of O-D, and 17% of B-D.

Sufficient time (50 ps) was allowed for the evolution cascade, and the cell was thermalized and relaxed after each impact. After the computational cell was prepared, with some fraction of accumulated D, its replicas were then bombarded with 3100 independent 5 eV D trajectories, orthogonal to the surface, and at random locations at the surface. The results reported here were therefore obtained as the average from 3100 D trajectories. The retention chemistry of D evolves at the end of a collision cascade when the impact particle is thermalized which allowed comparison with the experimental results at higher impact energies^33^. We carried out the analysis of the resulting chemistry after the final rest location of each D impact, by performing the nearest-neighbor (NN) calculation for each atom in the surface, defining the coordination number of each atom and the nearest neighbors (NN) bond lengths^33,47^. The histograms of the NN bond lengths, and their cumulative distributions, normalized with the total number of retained D atoms out of 3100 impacts for a particular D accumulation (shown for example of a pristine surface in Fig. 7) were used in derivation of the average NN bond lengths.

## Data Availability

The datasets generated and/or analyzed during the current study are available from the corresponding author on reasonable request.
