# Publisher Correction: Effect of deuterium irradiation on graphite boronized in the NSTX-U tokamak

**DOI:** 10.1038/s41598-020-59350-3

**Published:** 2020-02-05

**Authors:** F. Bedoya, J. P. Allain, F. J. Dominguez-Gutierrez, P. S. Krstic

**Affiliations:** 1 Plasma Science and Fusion Center, Massachusetts Institute of Technology, Cambridge, MA 02139 USA; 2 Department of Nuclear, Plasma, and Radiological Engineering, University of Illinois, Urbana, IL 61801 USA; 3 Max-Planck-Institute for Plasma Physics, Boltzmannstrasse 2, 85748 Garching, Germany; 4 Institute for Advanced Computational Science, Stony Brook University, Stony Brook, NY 11749 USA

Correction to: *Scientific Reports* 10.1038/s41598-019-38941-9, published online 21 February 2019

In the original version of this Article, the ORCID ID for J. P. Allain was omitted.

This error has now been corrected in the HTML and PDF versions of the Article.

